# The impact of neighborhood environment on the mental health: evidence from China

**DOI:** 10.3389/fpubh.2024.1452744

**Published:** 2025-01-07

**Authors:** Kaiqi Lei, Jianke Yang, Xiwang Ke

**Affiliations:** School of Humanities and Social Sciences, Xi’an Jiaotong University, Xi'an, China

**Keywords:** socioeconomic status, mental health, perception of environmental pollution, community social interaction, parallel mediation

## Abstract

**Introduction:**

The community environment is a significant social determinant affecting individual mental health.

**Purpose:**

This study explores the impact mechanisms and urban-rural heterogeneity in the relationship between socioeconomic status and individual mental health, focusing on community environmental perceptions and neighborhood interactions.

**Methods:**

This study used data from the 2021 Chinese General Social Survey (CGSS), selecting a sample of 1,974 respondents. First, a structural equation modeling (SEM) approach was employed for path analysis. Second, a bias-corrected nonparametric percentile bootstrap method was used to test for mediation effects and estimate confidence intervals. Finally, the heterogeneity of the mediation model across urban and rural communities was examined based on community type.

**Results:**

The results indicate that socioeconomic status is the primary determinant of mental health disparities. The mechanisms of environmental perception and social interaction play significant roles in bridging health disparities between social classes. Moreover, these mediating effects show considerable urban-rural heterogeneity. Specifically, the environmental perception mechanism has a stronger impact on rural residents, while social interaction mechanisms are more pronounced in urban communities.

**Discussion:**

The study emphasizes the importance of addressing environmental pollution and enhancing community social interactions as key strategies to reduce health disparities. Improving ecological governance and fostering community engagement, are essential for narrowing the health gap across socioeconomic groups.

## 1 Introduction

Health is an essential prerequisite for human development, a cornerstone of economic and social progress, and a symbol of a nation’s wealth and strength. China has consistently prioritized public health within its development strategy, with numerous initiatives aimed at improving national health. The CPC Central Committee and the State Council issued the “Healthy China 2030” Plan Outline, which emphasizes “placing health as a priority in development, and building and sharing health for all,” highlighting health’s role in promoting comprehensive human and socioeconomic development and proposing the development of “health care.” Simultaneously, the Outline sets the goal of establishing “healthy cities” and “healthy rural areas and towns.” The 14th Five-Year Plan for National Health, issued by the General Office of the State Council, acknowledges that while public health is steadily improving, Chinese residents still face a complex situation in which multiple health threats coexist and various factors influence health outcomes. Therefore, identifying the factors and processes that limit population health development at the theoretical level, while enhancing public health, is crucial for achieving Chinese-style modernization.

Link and Phelan (1) argued that socioeconomic status significantly influences individual health. Individuals from different socioeconomic backgrounds exhibit significant health disparities, which often worsen as the income gap between the wealthy and the impoverished expands; this social stratification of health is referred to as the “status syndrome” (2). Despite the fundamental role of socioeconomic status as a distal determinant of individual health, health issues are inevitably influenced by proximal factors. Consequently, it is crucial to thoroughly analyze the relationship between socioeconomic status and individual health, as well as investigate the mediating mechanisms through which socioeconomic status impacts health disparities.

Community serves as the foundation of an individual’s existence and plays a critical role in securing personal health. Previous research indicates that individuals dissatisfied with their communal environments are more likely to experience depression. Objective environmental factors within the community, such as polluted air, water, and noise, may exacerbate psychological stress and precipitate depression (3, 4). Interpersonal factors within the community may also influence residents’ health. The environment exerts a typical “neighborhood effect” on individual health at the community level (5). Neighborhood effects can be categorized into four primary types: social-interactive, social service, environmental, and geographical. The social-interactive and environmental mechanisms pertain to the effects of community social interactions and perceptions of pollution on individual health, respectively (6, 7). Established domestic studies have also examined the mediating mechanisms of health inequalities arising from an individual’s social status, encompassing not only proximal factors such as material resources, lifestyle, and psychosocial elements but also macro-environmental factors that contribute to illness and social contexts, emphasizing the importance of both macro (e.g., national) and micro (e.g., community) social environments (8–10).

This study was based on China’s 2021 General Social Survey (CGSS) data. From the standpoint of the community environment (community environment and community interpersonal environment), structural equation modeling was used to investigate how neighborhood effects affect health differentiation between classes and the urban–rural community heterogeneity of neighborhood effects. We sought to gain a deeper understanding of present health disparities through the lens of neighborhood effects and provide fresh theoretical insights and empirical experiences to improve China’s national health and promote equitable socioeconomic growth.

## 2 Literature review

### 2.1 The effect of socioeconomic status on individual mental health

Individual health stratification is a widespread sociological research topic. Academics have classified health-related factors based on their causal distance into three categories: distal (socioeconomic status and social environment), mid-range (community environment, neighborhood, and social support), and proximal (lifestyle) (11–13). Most scholars agree that socioeconomic position is an essential determinant of health (1). However, research on the intermediary mechanisms involved remains in its early stages. Although reducing health disparities between social classes remains a critical academic concern, studies indicate that there is no downward trend in health inequalities arising from socioeconomic status. Instead, the trend that individuals in higher socioeconomic status groups experience better health than those in lower socioeconomic status groups remains consistent across time and space (14).

Health is a multifaceted concept encompassing at least two dimensions: physical and mental health. According to the World Health Organization, mental health is an integral and essential component of overall health. Mental health encompasses not only the absence of mental disorders but also “the ability of an individual to recognize his or her abilities, cope with the normal stresses of life, work productively, and contribute to his or her community.” Socio-environmental factors are the most significant determinants of mental health. The incidence of mental disorders, particularly depression, among Chinese populations has shown a significant upward trend. More than 95 million people in China experience depression (15). Recent research indicates that mental health is more sensitive and responsive to changes in the social environment than physical health (16), suggesting that studying the health effects of social determinants through the lens of mental health may be more practical. The issues of socioeconomic status and health inequality are reflected in individuals’ psychological dimensions, as evidenced by the fact that individuals with higher socioeconomic status experience fewer mental illnesses, depressive episodes, and other negative emotions (17, 18). Based on this, the current study investigates the relationship between socioeconomic status and individual mental health, laying the foundation for subsequent tests of mediating mechanisms.

H1: Individual mental health is positively correlated with socioeconomic status.

### 2.2 Mediating effects of neighborhood effects

In recent years, scholars both domestically and internationally have extensively explored the mechanisms of neighborhood effects and concluded that the community environment shapes individual cognitive and behavioral patterns, both directly and indirectly, through a variety of mechanisms that profoundly impact individual mental health. The key influencing pathways identified include social-interactive, social service, environmental, and geographical mechanisms (19). This study focuses on community environmental issues, specifically investigating how environmental pollution and social interaction contribute to health disparities across social classes.

#### 2.2.1 Perception of environmental pollution

Environmental mechanisms refer to attributes such as environmental pollution within the community space where individuals reside, which may directly or indirectly affect residents’ mental health. These mechanisms are often used to explain the influence of community characteristics on individual health outcomes (7, 20). In exploring the relationship between socioeconomic status, subjective perceptions of environmental pollution, and mental health, theories such as environmental exploitation theory and environmental risk perception theory provide important theoretical frameworks.

Environmental exploitation theory is divided into absolute exploitation and relative exploitation. Absolute environmental exploitation emphasizes the direct impact of environmental pollution, considering it as an objective “exploiting force” that affects individuals’ physical and psychological health. Research indicates that long-term exposure to highly polluted environments, such as air and noise pollution, has a significant negative impact on individuals’ mental health, regardless of their socioeconomic status (36). In contrast, relative environmental exploitation theory focuses on the unequal exposure to environmental pollution due to socioeconomic disparities. It suggests that individuals with lower socioeconomic status are at a disadvantage in terms of exposure to environmental pollution, which is not only a result of the widespread presence of pollution but also due to their lower social status and lack of resources. Specifically, the lower an individual’s socioeconomic status, the higher their exposure to objective environmental pollution, which leads to a heightened perception of environmental pollution and a deterioration in mental health (21, 22).

Subjective environmental pollution perception refers to an individual’s subjective experience formed by their perception and psychological judgment of the surrounding environment and its changes, which plays an important mediating role in mental health. Environmental risk perception theory emphasizes that individuals’ perception of environmental pollution is not only dependent on the objective degree of pollution but also influenced by factors such as socioeconomic status and subjective cognitive biases (23). An increasing body of research shows that an individual’s subjective perception of environmental pollution does not always align with their actual objective exposure levels. Individuals with higher socioeconomic status typically possess better education, greater environmental knowledge, and stronger information access capabilities, which makes them more likely to perceive potential environmental risks when confronted with pollution. Even if individuals with higher socioeconomic status face lower levels of actual exposure, their environmental cognition and alertness lead to a stronger subjective perception of pollution. This subjective perception may cause “environmental anxiety” and prolonged psychological stress, which can have an adverse effect on their mental health (24, 25). In summary, by integrating environmental risk perception theory, the third hypothesis of this study is proposed:

H2: The higher an individual’s socioeconomic status, the greater their perception of environmental pollution, which negatively impacts their mental health.

#### 2.2.2 Community social interaction

The social-interactive mechanism refers to the endogenous social processes within a community, which are considered central to neighborhood effect theory (including social contagion, collective socialization, social networks, social cohesion, and social control) (20). Social science examines the behavioral consequences of individual social interactions, suggesting that individuals can enhance their social capital through interactions with neighbors, thereby obtaining benefits such as increased income and improved health. Studies indicate that individuals from lower socioeconomic backgrounds are more likely to rely on close-knit community ties to access these resources, exhibiting a greater inclination for social interaction within the community. In contrast, individuals from higher socioeconomic backgrounds tend to rely more on their personal abilities and external networks, often displaying lower levels of interpersonal interaction within the community (26, 27). While individuals with higher socioeconomic status can access additional resources by expanding their networks, this outwardly oriented network structure may reduce their social participation and interactions within the local community, thereby diminishing daily connections with neighbors. This “network separation” results in a lack of localized social support within the community, potentially leading to feelings of emotional isolation, which adversely affect mental health. Based on these findings, the third research hypothesis is proposed:

H3: Individuals with high socioeconomic status tend to participate in fewer socially engaged activities within society, which negatively impacts their mental well-being.

Hypotheses 2 and 3 posit that the community meso-environment can mitigate the health disadvantages of lower socioeconomic groups, safeguard the health of disadvantaged individuals, and bridge the health gap between classes, which will be empirically tested in this study.

### 2.3 Differences in urban–rural communities

Communities can be classified as rural or urban based on geographical factors. Urbanization has accelerated the division between China’s rural and urban populations, leading to disparities in health outcomes between the two groups (16). First, significant disparities exist in environmental contamination levels between rural and urban communities. Compared to rural communities, urban communities face more severe environmental pollution, including air pollution, water pollution, and noise. However, research indicates that the adaptation effect weakens the impact of external environmental stimuli on individual perception (28); when exposed to abundant informational stimuli, urban residents tend to prioritize other, more urgent issues, diminishing their perception of environmental pollution. Second, rural communities are often characterized by the intertwining of kinship and geographic relations, forming a “society of acquaintances,” whereas urban communities, based on geographic relations, are mobile, anonymous, and characterized by a “society of strangers.” Studies have shown that the closeness of social relationships influences expected outcomes for individuals, leading to variations in interactions between individuals of different social classes. Individuals with close social relationships are more likely to expect higher returns, whereas those who are more socially distant or “strangers” are less likely to experience such returns. There is no significant difference in interaction behaviors between high and low classes in rural communities when expected returns are high. However, in urban communities with low expected returns, there is a significantly larger difference in interaction behaviors between high and low classes (29). Thus, this study proposes the fourth research hypothesis:

H4: In urban and rural areas, the mediating effects of community social interaction and the perception of environmental pollution may vary. In particular, the mediating effect of community social interaction is significantly stronger in urban communities than in rural ones, and the mediating role of perceptions of environmental pollution is significantly stronger in rural communities than in urban ones.

### 2.4 The present study

**Figure 1:**
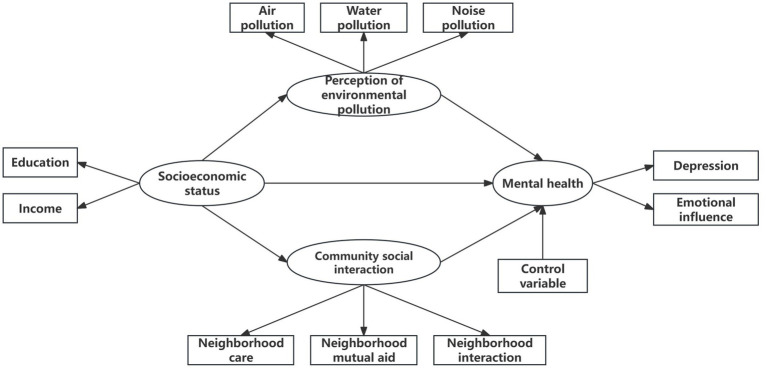
Conceptual model.

This study examined the relationship between socioeconomic status, neighborhood environment (perception of environmental pollution and community social interaction), and individual mental health (Figure 1). The first objective of this study was to test the effect of socioeconomic status on individual mental health and to verify the fundamental role of socioeconomic status. The second objective was to test the effect of socioeconomic status on individual mental health as mediated by the perception of environmental pollution and social interaction within the community. The final objective was to test the mediator hypothesis for variation between urban and rural communities.

## 3 Method

### 3.1 Data source

Data were obtained from the China General Social Survey (CGSS) conducted by the National Survey Research Centre at Renmin University of China. The CGSS is a pioneering and extensive academic survey program in China. It systematically and comprehensively gathers data from various levels of society, communities, households, and individuals. The CGSS serves as a multidisciplinary platform to collect economic and social data. This study employed the most recent data from 2021, encompassing 19 provinces, municipalities, and autonomous regions throughout the country. The overall sample size was 8,148 and there were 700 raw variables, ensuring that the data were representative and suitable for our research purposes. This study selected 15 variables from the questionnaire by thoroughly reviewing the literature and carefully analyzing the data from the CGSS (2021). The G environment module in the C thematic module includes variables related to environmental pollution perception and some observed variables of social interactions. The total sample size was 2,717. Because of large fluctuations in income data outliers, income-level outliers were removed. Outliers and missing values for the remaining 14 variables were replaced with their means and recorded. The final sample size was 1,974 participants.

### 3.2 Instruments

#### 3.2.1 Independent variable

Educational attainment and income levels are key determinants of socioeconomic status (SES) (30, 31). To assess these variables, participants were asked: “What is your highest level of education?” and “What was your total income for the last year (2020)?” Educational attainment was converted into years of schooling based on prior studies and categorized as follows: 0 = “no formal education,” 1 = “private school or literacy class,” 7 = “elementary school,” 8 = “junior high school,” 11 = “vocational high school, general high school, or junior college,” 13 = “college or university,” 16 = “bachelor’s degree,” and 19 = “postgraduate or higher.” Higher years of schooling indicated a higher level of education. Income data were adjusted by removing outliers and applying a logarithmic transformation for analysis.

#### 3.2.2 Dependent variable

Individual mental health assessment was examined using the self-assessed mental health items from the CGSS (2021) questionnaire: “In the past 4 weeks, how often have you felt depressed or down?” and “Due to emotional problems, you could not perform expected tasks or daily activities.” Individuals’ mental health status was recorded as 1 for “very unhealthy,” 2 for “relatively unhealthy,” 3 for “fair,” 4 for “relatively healthy,” and 5 for “very healthy.”

#### 3.2.3 Mediating variables

The mediating variables were perceptions of environmental pollution and community social interaction. The question “In the place where you live, how serious are the following problems?” was used to measure perceptions of environmental pollution. Three typical environmental perception problems were selected for this study: air, water, and noise pollution. The larger the value in the original data, the less severe the perception of environmental pollution. However, in this study, the three environmental pollution perception questions were reverse coded: 1 = “not serious at all,” 2 = “not too serious,” 3 = “more serious,” and 4 = “severe.” The higher the value, the more serious the individual’s perception of environmental pollution in the community.

Neighborhood interaction, care, and mutual aid are indicators of community interaction. The question selected for neighborhood interaction was “How often do you engage in social and recreational activities (e.g., visiting each other’s homes, watching TV together, eating together, playing cards) with your neighbors?” The options “never,” “once a year or less,” “several times a year,” “about once a month,” “several times a month,” “once or twice a week,” and “almost every day” were recoded 1 to 7 in order to create continuous variables. The greater the score, the more frequent the neighborhood interactions. The questions for neighborhood care and neighborhood mutual aid were “Neighbors around me care about each other” and “Neighbors are willing to help me when I am in need,” respectively. Responses were recoded as 1 = “totally disagree,” 2 = “disagree,” 3 = “neither agree nor disagree,” 4 = “agree,” and 5 = “completely agree.” Higher values indicated more frequent connections with the community.

#### 3.2.4 Control variable

To minimize potential confounding variables, we controlled for key demographic characteristics and urban–rural factors, including individual-level variables such as gender, age, political affiliation, and marital status, alongside urban–rural community characteristics. Gender, marital status, political affiliation, and community type are binary variables, whereas age is a continuous variable, calculated by subtracting the respondent’s year of birth from the survey year.

### 3.3 Analysis method

This study employed SPSS and AMOS statistical software to test the hypotheses by constructing structural equation models. The analytical process consisted of four main steps: first, structural equation modeling (SEM) was conducted to analyze the pathways; second, the bias-corrected nonparametric percentile bootstrap method, with 2,000 resamples, was applied to examine mediation effects and estimate confidence intervals; third, the heterogeneity of the mediation model was evaluated between urban and rural communities; and finally, the reliability and validity of the data were assessed.

## 4 Results

### 4.1 Descriptive statistics

The results of the independent samples t-test revealed significant differences between urban and rural residents across all measured variables. Descriptive statistics showed that the mean score for community interaction was higher in rural-level communities than in urban communities, whereas the mean scores for all other variables were lower in rural-level communities. These findings suggest that residents of rural-level communities engage in more frequent interactions, while urban residents report higher levels of environmental pollution perception, socioeconomic status, and mental health. The mean values for the three variables assessing perceptions of environmental pollution—air pollution (2.079/1.781), water pollution (2.007/1.858), and noise pollution (2.168/1.743)—ranged between “not serious at all” and “not too serious.” Among these, noise pollution was perceived more seriously than air and water pollution. However, the results indicate that participants did not consider environmental pollution to be a significant issue at present. Regarding community social interaction, the means for neighborhood mutual aid (3.895/4.173) and neighborhood care (3.792/4.130) were similar, suggesting relatively frequent social interactions. The mean for neighborhood interaction (3.438/4.124) indicated that interactions typically occur “several times a year” to “about once a month.” In terms of socioeconomic status, the mean level of educational attainment (11.065/7.479) corresponded to the middle school education category, while the mean income was approximately 100,000 yuan. For mental health measures, the mean values for depressed mood (4.053/3.836) and emotional impact (4.237/3.996) were classified as “relatively healthy.” Overall, participants were considered to have relatively good psychological health (Table 1).

**Table 1:** Distribution of core variables and differences between urban and rural areas.

Variables		Urban (*n* = 1,149)	Rural (*n* = 825)	*t*	*p*
Socioeconomic status	Education	11.065 (4.249)	7.479 (4.061)	18.981	0.000
	Income	10.562 (1.138)	9.381 (1.363)	20.308	0.000
Perception of environmental pollution	Air pollution	2.079 (0.750)	1.781 (0.778)	8.535	0.000
	Water pollution	2.007 (0.763)	1.858 (0.836)	4.065	0.000
	Noise pollution	2.168 (0.802)	1.743 (0.815)	11.500	0.000
Community social interaction	Neighborhood care	3.792 (0.918)	4.130 (0.763)	−8.905	0.000
	Neighborhood mutual aid	3.895 (0.827)	4.173 (0.742)	−7.841	0.000
	Neighborhood interaction	3.438 (2.187)	4.124 (2.180)	−6.885	0.000
Mental health	Depression	4.053 (1.010)	3.836 (1.084)	4.506	0.000
	Emotional influence	4.237 (0.872)	3.996 (1.027)	5.478	0.000

Sample means are outside parentheses and standard deviations are in parentheses.

### 4.2 Model fitness checker and path analysis

We introduced two mediating variables—perception of environmental pollution and community social interaction—into our model. This allowed us to create a parallel-mediated structural equation model that included the latent variables. Model parameters were estimated using the maximum likelihood method. The model’s fit indices were determined to be *x^2^*/df = 4.347, RMSEA = 0.041, SRMR = 0.044, CFI = 0.968, and TLI = 0.952. Based on the established criteria for evaluating model fit, which are *x^2^*/df < 5, CFI > 0.90, TLI > 0.90, RMSEA<0.08, and SRMR≤0.05/0.08 (32), each fitting index of the structural equation model developed in this study fell within an acceptable range. This suggests that the model had a good fit.

**Figure 2:**
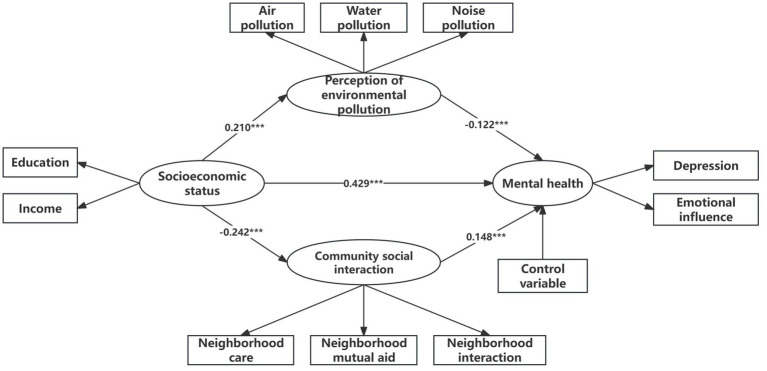
Results of the standardized path analysis. Statistical significance levels: *p < 0.05, **p < 0.01, ***p < 0.001.

The standardized path analysis results, presented in Table 2, indicate a significant positive correlation between socioeconomic status and individual mental health at a significance level of 0.001. Moreover, the mediating effects of environmental pollution perception and community interaction are also significant. Therefore, Hypotheses 1, 2, and 3 are supported by the findings (Figure 2).

**Table 2:** Results of the standardized path analysis.

Path	Coefficient	S.E	C.R	*p*
Socioeconomic status→Perception of environmental pollution	0.210	0.021	7.367	0.000
Perception of environmental pollution→Mental health	−0.122	0.033	−3.831	0.000
Socioeconomic status→Community social interaction	−0.242	0.024	−7.100	0.000
Community social interaction→Mental health	0.148	0.036	4.450	0.000
Socioeconomic status→Mental health	0.429	0.047	6.965	0.000

### 4.3 Test for the bootstrap mediation effect

Table 3 presents the bootstrap mediation effect test. Based on a good model fit, the bias-corrected nonparametric percent bootstrap test in the AMOS software was used to estimate confidence intervals and conduct mediation effect tests by repeating the sampling 2,000 times, where indirect effects were significant if the 95% confidence intervals did not include zero. The results indicated that the confidence intervals for the direct, mediated, and total effect paths of the model did not include zero, suggesting that perceptions of environmental pollution and community social interaction partially mediate the relationship between socioeconomic status and individual mental health.

**Table 3:** Results of the bootstrap mediation effect.

Effect	Path	Efficiency value	95% Confidence interval(CI)
Direct effect	Socioeconomic status→Mental health	0.429	[0.311,0.548]
Mediation effect	Socioeconomic status→Perception of environmental pollution→Mental health	−0.026	[−0.044,-0.012]
	Socioeconomic status→Community social interaction→Mental health	−0.036	[−0.056,-0.021]
Total effect		0.367	[0.251,0.483]

Thus, Hypotheses 1, 2, and 3 were supported. Socioeconomic status negatively impacted individual mental health through perceptions of environmental pollution (−0.026) and community social interaction (−0.036). In contrast, the direct effect of socioeconomic status on mental health was positive (0.429), which was greater than the total effect (0.367). This suggests that the mediators had an inhibitory role; that is, perceptions of environmental pollution and community social interaction acted as inhibitory mediators, partially reducing the mental health disparities associated with socioeconomic status.

### 4.4 Test for differences in urban–rural communities

This study investigated whether the mediating effects of environmental pollution perceptions and community social interactions vary across urban and rural areas. The test results are presented in Table 4. Parallel mediation effect models examining the relationship between socioeconomic position and individual mental health were independently assessed for the urban and village groups. The results indicated that the fitted indicators in the urban community were as follows: *x^2^*/df = 3.023, RMSEA = 0.042, SRMR = 0.046, CFI = 0.963, and TLI = 0.946. Similarly, the fitted indicators in the rural community were as follows: *x^2^*/df = 1.842, RMSEA = 0.032, SRMR = 0.044, CFI = 0.979, and TLI = 0.970. The results indicated that all measured indicators for both urban and rural communities fell within the permissible range for making comparisons between groups. Next, the goodness of fit of the unrestricted model (baseline model) for different community types and that of the model after equalizing the path coefficients of the restricted structure were examined. The results showed that both models’ goodness of fit fell within reasonable bounds: the unrestricted model (baseline model) was *x^2^*/df = 2.432, RMSEA = 0.027, CFI = 0.970, and TLI = 0.956, and the restricted models were *x^2^*/df = 2.498, RMSEA = 0.028, CFI = 0.967, and TLI = 0.956. Furthermore, the fit indices of the two models showed substantial differences (*p* = 0.001). The rejection of the equivalence hypothesis between the baseline and restriction models suggests a notable distinction between them.

**Table 4:** Tests for mediating effects of urban–rural heterogeneity.

Effect	Path	Urban		Rural	
		Efficiency value	95%CI	Efficiency value	95%CI
Direct effect	Socioeconomic status→Mental health	0.205	[0.064, 0.337]	0.684	[0.402, 1.071]
Mediation effect	Socioeconomic status→Perception of environmental pollution→Mental health	−0.007	[−0.023, 0.001]	−0.026	[−0.054, −0.008]
	Socioeconomic status→Community social interaction→Mental health	−0.028	[−0.053, −0.012]	−0.017	[−0.043, −0.002]
Total effect		0.170	[0.028, 0.300]	0.640	[0.366, 1.024]

**Figure 3:**
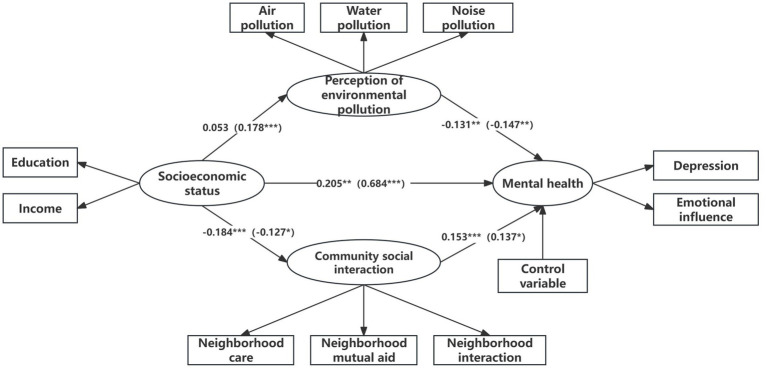
Tests for mediating effects of urban–rural heterogeneity. *n* = 1,974, *n*_urban_ = 1,149, *n*_rural_ = 825; regression coefficients for residents of urban communities are outside parentheses and those for residents of rural communities are inside parentheses. Statistical significance levels: *p < 0.05, **p < 0.01, ***p < 0.001.

We also tested the mediating effect of perceptions of environmental pollution and community social interaction between individual socioeconomic status, mental health, and community differences. Table 4 presents the results of the mediating effect tests. The findings showed differences in the mediating effects of perceptions of environmental pollution and community social interaction between urban and rural communities. In terms of the presence or absence of mediating effects, both mediating pathways existed in rural communities, while the mediating pathway of perceived environmental pollution did not exist in urban communities (Figure 3). The absolute value of the social interaction effect (−0.028) was higher in urban communities than in rural ones (−0.017), but the absolute value of the environmental pollution perception effect (−0.007) was much lower than that in rural communities (−0.026). The study’s findings supported Hypothesis 4, that individual environmental pollution perceptions are mediated significantly more in rural communities than in urban ones, and community social interaction is mediated significantly more in urban communities than in rural ones, indicating that the mediating variable (with a negative effect value) acts as a suppressor.

## 5 Discussion

### 5.1 Main results

This study aims to examine the influence of social class differences on individual mental health through the neighborhood interaction mechanism, which includes environmental pollution perception and community interaction, and to analyze the variations in this mechanism across urban and rural areas. The results demonstrate that socioeconomic status significantly influences individual mental health, and that environmental pollution perception and community interaction, as mediating variables, exert an inhibitory effect between socioeconomic status and mental health. Specifically, while socioeconomic status positively affects individual mental health, environmental pollution perception and community interaction help mitigate the mental health disparities resulting from social class differences. Furthermore, the impact of this mechanism exhibits significant heterogeneity between urban and rural communities: the effect of environmental pollution perception is more pronounced among rural residents, whereas the community interaction mechanism is primarily effective in urban communities.

### 5.2 Interpretation

#### 5.2.1 Socioeconomic status and mental health

There is a significant positive relationship between socioeconomic status and individual mental health, with higher socioeconomic status being associated with better mental health, a finding that is consistent with prior research. From the perspective of resource acquisition and distribution, individuals with higher socioeconomic status typically possess greater economic resources, which can significantly alleviate material stress and uncertainty in their lives. Furthermore, a stable economic situation not only reduces the risk of anxiety and depression due to insufficient income but also offers additional options and security for coping with unforeseen events.

#### 5.2.2 Mediating effects of environmental pollution perception and community social interaction

The neighborhood effect is a crucial mechanism for addressing the health disparities between different social classes driven by socioeconomic status. Educational attainment and individual income levels, as forms of social capital, reflect a person’s knowledge, cognitive abilities, and capacity to access resources, thus influencing mental health through environmental perceptions. Perceptions of environmental pollution partially mediate the relationship between socioeconomic status and individual mental health, yielding a negative effect value of −0.026. Individuals with higher socioeconomic status tend to possess greater awareness of environmental pollution, which negatively impacts their mental health. This phenomenon can be explained from several perspectives. (1) Individuals with higher socioeconomic status typically have higher levels of education and stronger information acquisition abilities, allowing them to access more information, research, and policies related to environmental pollution, thus increasing their sensitivity to environmental issues (23). (2) Individuals with higher socioeconomic status often have broader social networks, making them more likely to engage in public discussions and activities related to environmental protection, which in turn raises their awareness of environmental issues. However, this heightened sensitivity to environmental pollution may adversely affect individuals’ mental health. Research suggests that individuals with higher socioeconomic status may experience heightened anxiety, fear, and uncertainty when confronted with potential environmental risks, which may ultimately result in mental health issues such as anxiety and depression.

Community social interaction shows a negative mediating effect of −0.036 between socioeconomic status and individual mental health. Specifically, individuals with higher socioeconomic status engage less frequently in social interactions within their communities, which negatively affects their mental health. This phenomenon may arise from the tendency of individuals with higher socioeconomic status to rely more heavily on their own abilities and external networks. Such outward-oriented network structures reduce their participation and interaction within local communities, diminishing daily contact with neighbors and thus negatively impacting mental health (26, 27). Additionally, individuals with higher socioeconomic status may experience a certain degree of “social isolation” due to their privileged status, further undermining their sense of community belonging and emotional connection, which, in turn, exacerbates the adverse effects on mental health.

Research indicates that strong neighborhood interactions significantly promote mental health, primarily through two mechanisms: (1) Community social networks: Neighborly relations facilitate the dissemination of health information and access to material and emotional support, thus improving individual physical and mental well-being (33). (2) Community cohesion: Neighborly relationships, as a key indicator of community cohesion, provide valuable social connections and mutual respect, which effectively enhance individuals’ psychological well-being. Overall, a supportive interactive environment not only provides essential support for mental health but also exerts a positive influence on individual psychological well-being (34).

#### 5.2.3 Urban and rural community differences

The mediating effect of environmental pollution perception differs significantly between urban and rural communities. While it is not significant in urban communities, it exerts a notable influence in rural communities. Specifically, the inhibitory effect of individual perceptions of environmental pollution is significantly stronger in rural-level communities (−0.026) compared to urban communities (−0.007). This discrepancy can be attributed to the fact that urban communities often face more severe environmental pollution issues, such as air pollution, water pollution, and noise. Prolonged exposure to these conditions may trigger an “adaptation effect,” diminishing the impact of external environmental stimuli on individuals’ subjective perceptions. Moreover, urban residents have access to more diverse channels of information and multifaceted social support networks, which can effectively alleviate the psychological stress caused by “environmental anxiety,” thereby mitigating the adverse effects of environmental pollution on mental health (35). In contrast, rural residents, when confronting environmental pollution issues, often rely on relatively limited social support networks, making it difficult to obtain sufficient emotional or resource-based support, and are consequently more vulnerable to psychological problems arising from environmental pollution.

The community social interactive inhibitory effects were significantly higher in urban communities (−0.028) than in rural ones (−0.017). This study suggests that rural-level communities, characterized by strong relationships based on geographic and kinship ties, have closer social distances and higher expectations of reciprocal interactions. As a result, the difference in interaction behaviors between higher and lower socioeconomic classes is less pronounced, leading to a relatively weaker inhibitory effect of social interaction on health disparities caused by socioeconomic status in rural communities. In contrast, urban communities, shaped by geographic relationships, are marked by greater social distance, less frequent connections, and lower expectations of reciprocal returns. Consequently, the difference in interaction behaviors between higher and lower socioeconomic classes is more significant, resulting in a stronger inhibitory effect of social interaction on health disparities in urban communities.

### 5.3 Reliability and validity

The study employed Cronbach’s alpha coefficient to assess reliability; the higher the alpha, the better the reliability and internal consistency of the questionnaire. Cronbach’s *α* coefficients for each latent variable exceeded 0.65, suggesting adequate reliability of the scale. Convergent and discriminant validities were the primary methods used to test validity, which was measured using two indicators: average variance extracted (AVE) and combined reliability (CR). As shown in Table 5, the AVE values of each variable were above 0.5 and the CRs were approximately 0.7, indicating that the variables had a certain degree of convergent validity. Discriminant validity was measured by comparing the square root of the latent variable AVE with the correlation coefficient between the latent variable and other latent variables. As shown in Table 5, the correlation coefficients between any variables in the table were smaller than the square root of each variable’s AVE, indicating good discriminant validity between the question items measuring different variables.

**Table 5:** Results of reliability and validity tests (*n* = 1,974).

Variables	1	2	3	4	AVE	CR	Cronbach’s α
1.Socioeconomic status	0.728				0.531	0.693	0.692
2.Perception of environmental pollution	0.170	0.724			0.524	0.763	0.752
3.Community social interaction	−0.213	−0.143	0.730		0.532	0.745	0.682
4.Mental health	0.216	−0.057	−0.004	0.710	0.504	0.668	0.663

Values on the diagonal are the square roots of the AVE and values below the diagonal are the correlation coefficients between the variables.

## 6 Limitations and future directions

First, this study employs survey data for empirical research, with many of the variables being subjective, such as environmental pollution perception, community interaction, and individual health. These subjective variables may introduce bias into the results. Future studies could incorporate objective variables for measurement. Second, due to data constraints, the measurement of environmental pollution perception used in this study may extend beyond the community level and is relatively simplistic in its dimensions. Future research should adopt a multi-dimensional approach to assess the measurement standards for environmental pollution perception and community interactions. Third, this study employs a cross-sectional design, which limits the causal interpretability of the findings. Specifically, cross-sectional research can only reveal correlations between socioeconomic status, environmental pollution perception, community interaction, and individual mental health at a specific point in time, without allowing for direct inference of causal relationships between these variables. Future research could consider using longitudinal designs or experimental methods to track the same group of individuals over time, facilitating a more accurate assessment of the long-term effects of socioeconomic status, environmental pollution perception, and community interaction on mental health, thereby providing stronger evidence for causal inference. Fourth, due to limitations in the research data, the analysis of neighborhood effects as a mediating mechanism only considered community environment and social interaction mechanisms. The analysis of the mechanism is incomplete and should incorporate additional factors, such as social services and geographical influences.

## 7 Conclusion

This study situated individuals in specific microsocial environments through the lens of environmental embeddedness. It investigated the mechanisms of influence and urban–rural heterogeneity between socioeconomic status and individual mental health in the perception of environmental pollution and community social interaction. This study found that socioeconomic status is a crucial factor influencing individual mental health disparities. At the same time, environmental perception and social interaction in the community play essential roles in bridging the health divide between classes with significant urban–rural heterogeneity. This finding adds to the recognition of health inequalities in modern society. Furthermore, this study holds practical significance as it presents novel theoretical discoveries and empirical experiences to improve national health and promote balanced socioeconomic growth in China.

## Data Availability

The datasets presented in this study can be found in online repositories. The names of the repository/repositories and accession number(s) can be found below: http://cgss.ruc.edu.cn/.
